# An energy landscape approach reveals the potential key bacteria contributing to the development of inflammatory bowel disease

**DOI:** 10.1371/journal.pone.0302151

**Published:** 2024-06-17

**Authors:** Kaiyang Zhang, Shinji Nakaoka

**Affiliations:** 1 Graduate School of Life Science, Hokkaido University, Sapporo, Japan; 2 Faculty of Advanced Life Science, Hokkaido University, Sapporo, Japan; Mayo Clinic Rochester, UNITED STATES

## Abstract

The dysbiosis of microbiota has been reported to be associated with numerous human pathophysiological processes, including inflammatory bowel disease (IBD). With advancements in high-throughput sequencing, various methods have been developed to study the alteration of microbiota in the development and progression of diseases. However, a suitable approach to assess the global stability of the microbiota in disease states through time-series microbiome data is yet to be established. In this study, we have introduced a novel Energy Landscape construction method, which incorporates the Latent Dirichlet Allocation (LDA) model and the pairwise Maximum Entropy (MaxEnt) model for their complementary advantages, and demonstrate its utility by applying it to an IBD time-series dataset. Through this approach, we obtained the microbial assemblages’ energy profile of the whole microbiota under the IBD condition and uncovered the hidden stable stages of microbiota structure during the disease development with time-series microbiome data. The *Bacteroides*-dominated assemblages presenting in multiple stable states suggest the potential contribution of *Bacteroides* and interactions with other microbial genera, like *Alistipes*, and *Faecalibacterium*, to the development of IBD. Our proposed method provides a novel and insightful tool for understanding the alteration and stability of the microbiota under disease states and offers a more holistic view of the complex dynamics at play in microbiota-mediated diseases.

## Introduction

The microbiota in humans plays a crucial role in maintaining health and well-being, with varying composition in different body sites, including the mouth, vagina, skin, and notably, the intestinal tract [1]. It has also been dubbed as a “forgotten organ” due to its collective and complex metabolic activity [2]. Bowel dysbiosis, an imbalance in the composition of the microbiota, has been linked to numerous diseases, including gastrointestinal disorders, such as inflammatory bowel disease (IBD) [3]. A comprehensive understanding of the impact and mechanisms of microorganism-host interactions is essential for diagnosing and treating associated diseases.

Having the recognition of the complexity of the pathogenic mechanisms of the gut microbiota, current microbiome research performs community-level and muti-omics analysis to uncover the association between gut microbiota and diseases, including the IBD study [4]. Current studies elucidated the heterogeneity of gut microbiota in the IBD development stages and categorization [5, 6], suggesting the value of the analysis of time-series data from longitude to capture the dynamic feature of the alteration of the microbiome during the disease pathogenesis. However, it is still a challenge for conventional methods to uncover the hidden microbiota structure from the time-series data.

We carried out energy landscape analysis combining the Latent Dirichlet Allocation (LDA) model and pairwise Maximum Entropy (MaxEnt) model to IBD gut microbiome dataset. Our results show multiple stable structure patterns in the Crohn’s disease patient, characterized by the alteration of genus *Bacteroides*, implies the key role of *Bacteroides* in shaping the dysbiosis stages and their transition in the development of IBD.

The Latent Dirichlet Allocation model is a widely applied unsupervised machine learning method in natural language processing (NLP). It models text through a three-level hierarchical Bayesian model, with “topic-word” and “document-topic” multinomial distributions and a Dirichlet prior [7]. In the context of microbial abundance profiles, the LDA model can identify “microbial assemblages” by grouping taxa according to their co-occurrence features [8, 9], similar to the “topics” in NLP studies. Additionally, the pairwise MaxEnt model provides a second-order maximum entropy model that captures a single node’s firing rates and the pairwise interactions in the biological system, assuming higher-order interactions are not crucial and set aside [10, 11]. This model has been demonstrated to accurately describe neural systems using time-series MRI data [10, 12]. The pairwise MaxEnt model has been introduced to study the stability of microbial community by Kenta et al. [13], assuming the components have pairwise interactions akin to neuronal activity.

**Fig 1:**
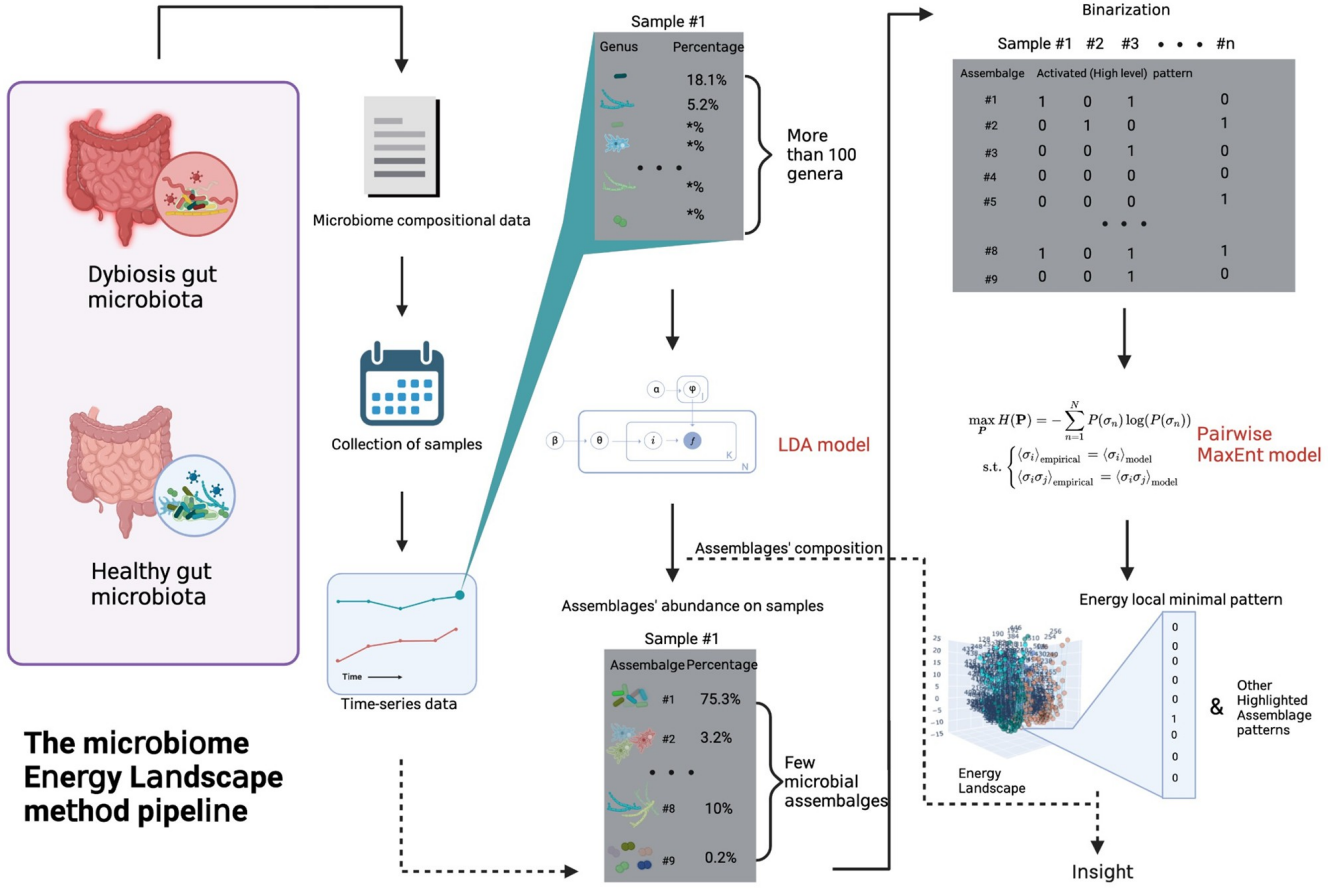
The scheme of microbiome Energy Landscape method.

In this study, we propose the LDA model to cluster the microbial abundance profile into a few microbial assemblages according to co-occurrence features and then the pairwise MaxEnt model to calculate an “energy” profile for all potential activity patterns of microbial assemblages. Finally, the derived Energy Landscape depicts the overall stability of assemblage patterns and the relationship among them under specific health conditions. We investigated the stable assemblage patterns under the conditions and discussed the key microbial elements that may contribute to shaping the intermediate stages of dysbiosis (Fig 1).

## Materials and methods

### Ethics statement

The data used in this study are all available in the public domain(The Integrative Human Microbiome Project (iHMP)(NIDDK U54DE023798)) [14], ethical approval is not applicable to this study.

### Metagenomic time-series dataset

We have used the dataset from the Onset of Inflammatory Bowel Disease (IBD) of The Integrative Human Microbiome Project (iHMP)(NIDDK U54DE023798) [14]. The dataset contains taxonomic profiles of fecal samples’ 16S rDNA sequencing results from participants. These taxonomic profiles of each participant were collected repeatedly during the study period. Here, we selected each participant’s first ten successive time-series samples and excluded the participants with less than ten samples. Finally, the sample size comprised 1300 samples collected from 130 participants, each contributing 10 samples. Several participants were diagnosed with two major types of IBD: Crohn’s Disease (CD) and Ulcerative Colitis (UC), while the remaining participants without IBD (non-IBD) served as control.

### The sample size

Table 1 presents the information of classes in this study. In the LDA modeling step, only the first samples from the time-series samples of each participant were used as the input (*N* = 130) to avoid the bias resulting from the homogeneity of the microbial community’s composition from the same participant. After learning the parameter ***φ***_*i*_ from the LDA model, the model was applied to the 780 samples (as detailed below) as the next step’s input. In the pairwise MaxEnt modeling step, the modeling was conducted separately for three disease types. To facilitate comparisons, balanced input in the three classes (each class for *N* = 26 × 10 = 260), consisting of 780 samples in total, were chosen for modeling execution on each class.

**Table 1:** The classes in the study. The numbers without brackets represent the number of participants, and in bracket represent the number of samples.

Disease types	CD	UC	non-IBD
Modeling steps			
LDA modeling	65(65)	38(38)	27(27)
Pairwise MaxEnt modeling	26(260)	26(260)	26(260)

### Latent Dirichlet Allocation modeling

The Latent Dirichlet Allocation model is a generative statistical model applied to observations with unobserved latent attributes. According to the principle of the LDA model, the microbiota, or microbial community, is comprised of a series of single “occurrence-event” (hereafter referred to as occurrence). An occurrence is defined as the solitary presence of a taxonomic unit. Each occurrence belongs to a latent attribute: microbial assemblage.

Hence the generative process of a microbial community, with *I* potential microbial assemblages and *F* genera in *N* samples, can be assumed as follows:

(1) The *k*-th occurrence in the sample *n*, among *N* samples, *O*_*nk*_, has a latent assemblage attribute *i* which follows the multinomial distribution with parameters ***θ***_*n*,*n*∈(1,…,*N*)_,
  $$I_{n}∼Multinomial\left(\theta_{n,n\in (1,\ldots ,N)}\right).$$
  Sampling from the distribution assigns the assemblage attribute *i* to the occurrence;
(2) The taxonomic unit of the occurrence, genus *f*, given the assemblage *i* follows a multinomial distribution with parameters ***φ***_*i*,*i*∈(1,…,*I*)_,
  $$F_{i}∼Multinomial\left(\varphi_{i,i\in (1,\ldots ,I)}\right).$$
  After sampling from the distribution, one occurrence with genus *f* in sample *n* is set.
(3) The (1)-(2) process repeats in *O*_*n*(*k*+1)_, and ultimately, the occurrences combine to form the microbial community in sample *n*.

Notably, the parameter of multinomial distributions of *I*_*n*_, vector $\theta_{n}=\left(\theta_{n_{1}},\ldots ,\theta_{n_{I}}\right)$, follows the Dirichlet distribution with prior parameter ***β***_*n*_,

$$\theta_{n}∼Dirichlet\left(I,\beta_{n}\right).$$

The parameter of multinomial distributions of *F*_*i*_, vector $\varphi_{i}=\left(\varphi_{i_{1}},\ldots ,\varphi_{i_{F}}\right)$, follows the Dirichlet distribution with prior parameter ***α***_*i*_,

$$\varphi_{i}∼Dirichlet\left(F,\alpha_{i}\right).$$

**Fig 2:**
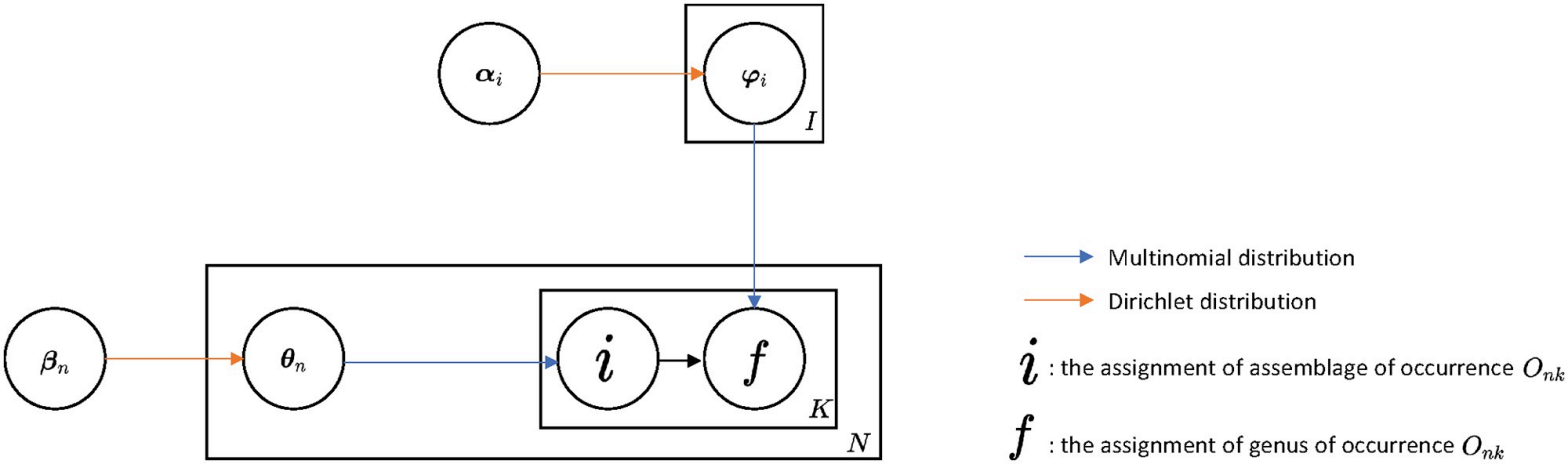
Graphical model of the LDA model.

Fig 2 depicts the aforementioned generative process.

Based on multinomial distribution, parameter ***θ***_*n*_ and ***φ***_*i*_ can represent the probability of the occurrences of assemblages given the sample *n* (*p*(*i* ∣ ***θ***_***n***_) = *θ*_*ni*_) and the probability of the occurrences of genera given the assemblage *i* (*p*(*f* ∣ ***φ***_***i***_) = *θ*_*if*_), respectively. Thus, ***θ*** and ***φ*** can be regarded as the “abundance” of *I* assemblages in a specific sample and the weight of *F* genera in one specific assemblage, respectively.

After parameter estimation of ***θ***_*n*_ and ***φ***_*i*_, the *F*-dimension original genera abundance profile was reduced to *I*-dimension assemblages abundance profile, which was processed to the following Maximum Entropy Modeling.

Here, we selected *I* = 9 as the number of assemblages that reduces the computational cost of pairwise Maximum Entropy modeling and maintains interpretability (see also discussion). And for the reason mentioned in the previous section, we fit the LDA model to a few samples(*N* = 130) to fix the composition of assemblages ***φ*** and applied the model to all samples(*N* = 780) to obtain the abundance of assemblages ***θ*** in all samples. The LDA modeling was performed using Python sklearn.decomposition.LatentDirichletAllocation package [15–17], the statistical analysis was performed by Python Scipy package [18].

### Pairwise Maximum Entropy modeling

We fit the pairwise Maximum Entropy model according to the manners in its previous applications for neuroscience [11, 12, 19]. In the pairwise MaxEnt model, the objective was to maximize the information entropy of probability distribution under the Maximum Entropy Principle and fit the model’s strength of individual assemblage and pair interactions to empirical data, represented by the constraints of 〈***σ***_*i*_〉 and 〈***σ***_*i*_***σ***_*j*_〉, respectively. 〈***σ***_*i*_〉 and 〈***σ***_*i*_***σ***_*j*_〉 are defined as follows:

$${\left\langle \sigma_{i}\right\rangle }_{empirical}=\frac{1}{N}\sum_{n=1}^{N}\sigma_{i_{n}},{\left\langle \sigma_{i}\right\rangle }_{model}=\sum_{\sigma^{\prime }}\sigma_{i}^{\prime }P\left(\sigma^{\prime }\right),$$

where $\sigma_{i_{n}}=\pm 1$ is the occurrence state of the *i*-th assemblage on the *n*-th sample;

$${\left\langle \sigma_{i}\sigma_{j}\right\rangle }_{empirical}=\frac{1}{N}\sum_{n=1}^{N}\sigma_{i_{n}}\sigma_{j_{n}},{\left\langle \sigma_{i}\sigma_{j}\right\rangle }_{model}=\sum_{\sigma^{\prime }}\sigma_{i}^{\prime }\sigma_{j}^{\prime }P\left(\sigma^{\prime }\right),$$

where *i* and *j* represent two different assemblages, *empirical* represents the empirical results and *model* represents the expected value given by the model.

The whole model can be derived using

$$\underset{P}{\text{max}}\;H(\text{P})=-\sum_{n=1}^{N}P\left(\sigma_{n}\right)\text{log}\left(P\left(\sigma_{n}\right)\right)$$

$$s.t.\{\begin{array}{c} {\left\langle \sigma_{i}\right\rangle }_{empirical}={\left\langle \sigma_{i}\right\rangle }_{model}\\ {\left\langle \sigma_{i}\sigma_{j}\right\rangle }_{empirical}={\left\langle \sigma_{i}\sigma_{j}\right\rangle }_{model} \end{array}.$$

The pairwise MaxEnt model illustrates the probability of assemblage patterns ***σ*** to occur in the following distribution:

$$P\left(\sigma |h,g\right)=\frac{\text{exp}[-E(\sigma |h,g)]}{\sum_{\sigma^{\prime }}\text{exp}\left[-E\left(\sigma^{\prime }|h,g\right)\right]},$$

where

E(σ∣h,g)=-∑i=1Ihiσi-12∑i=1I∑j=1i≠jIgijσiσj,

The ***h*** and ***g*** are the parameters that need to be estimated from the data, representing the tendency to the occurrence of one assemblage and the interaction between two assemblages, respectively. Positive and negative values of ***g*** are interpreted as promotional and inhibitory interactions, respectively.

We estimated the parameters through the maximum-likelihood method [19]. Here, we solved

$$\left(h,g\right)=\underset{h,g}{\text{argmax}}\;L\left(h,g\right),$$

where L(h,g) is the likelihood function given by

$$L\left(h,g\right)=\prod_{n=1}^{n_{\text{max}}}P\left(\sigma_{n}|h,g\right).$$

The likelihood was maximized by updating ***h*** and ***g*** in the gradient ascent scheme till convergence:

$$\left\{ \begin{array}{c} h_{i}^{\text{new}\;}-h_{i}^{\text{old}\;}=\frac{\epsilon }{n_{\text{max}}}\frac{\partial }{\partial h_{i}}\text{log}\;L\left(h,g\right)=\epsilon \left({\left\langle \sigma_{i}\right\rangle }_{\text{empirical}\;}-{\left\langle \sigma_{i}\right\rangle }_{\text{model}\;}\right)\\ g_{ij}^{\text{new}\;}-g_{ij}^{\text{old}\;}=\frac{\epsilon }{n_{\text{max}}}\frac{\partial }{\partial J_{ij}}\text{log}\;L\left(h,g\right)=\epsilon \left({\left\langle \sigma_{i}\sigma_{j}\right\rangle }_{\text{empirical}}-{\left\langle \sigma_{i}\sigma_{j}\right\rangle }_{\text{model}\;}\right) \end{array},\right.$$

where *new* and *old* represent the values after and before a single updating step, relatively, and *ϵ* > 0 is a constant controlling the step size.

The Pairwise Maximum Entropy modeling was performed using Python Numpy package [20].

### Definition of the occurrence state in assemblage pattern

The pairwise MaxEnt model required a binary input. Here, we defined the assemblage pattern as ***σ***, where the value of each microbial assemblage ***σ***_*i*,*i*∈(1,…,*I*)_ was assigned either 1 or -1 according to its “occurrence state.” This state represents whether the specific microbial assemblage has a relatively high abundance on a sample. Recall that the assemblage’s abundance in each sample is assigned by the parameter ***θ***_*n*_ given by the LDA model if *i*-th assemblage of *n*-th sample has a higher probability parameter than that of *m*-th sample, $\theta_{i_{n}}>\theta_{i_{m}}$, given by the LDA model. We considered that the *i*th assemblage shows higher abundance on the microbial community of *n*-th sample than *m*-th sample.

Here, a threshold was set to define the relatively high abundance or “activated” assemblage for binarization. We assigned the occurrence state ***σ***_*i*_ under the following rule:

$$\sigma_{i_{n}}=\{\begin{array}{c} +1,\;\text{if}\;\theta_{n_{i}}\;\text{is}\;\text{greater}\;\text{than}\;\text{the}\;\text{upper}\;\text{25th}\;\text{percentile}\;\text{of}\;\left\{\theta_{i}\right\}\\ -1,\;\text{if}\;\theta_{n_{i}}\;\text{is}\;\text{less}\;\text{than}\;\text{the}\;\text{upper}\;\text{25th}\;\text{percentile}\;\text{of}\;\left\{\theta_{i}\right\} \end{array},$$

where $\theta_{n_{i}}$ is the abundance of *i*th assemblage in *n*th sample given by the LDA model and {***θ***_*i*_} is the set of the abundance of *i*th assemblage in all samples of a class. The occurrence state of assemblage in a microbial community corresponds to the binary spike state of a single neuron in Schneidman’s study, which assigns the response of the neuron in a binary state of 1 (spike) and 0 (not spike) [11]. We then integrated two models through this definition by transferring the output from LDA modeling ***θ*** to the binary input for pairwise MaxEnt modeling ***σ***.

### Energy Landscape

The distribution we obtained from the pairwise MaxEnt model had the form of the Boltzmann distribution in statistical mechanics:

$$p_{i}=\frac{1}{Q}\text{exp}\left[-\varepsilon_{i}/\left(kT\right)\right]=\frac{\text{exp}[-\varepsilon_{i}/\left(kT\right)]}{\sum_{j-1}^{M}\text{exp}\left[-\varepsilon_{j}/\left(kT\right)\right]},$$

where *ε*_*i*_ is the energy of the system at state *i*, *k* the Boltzmann’s constant, and *T* the temperature [11]. Recalling the distribution of assemblage pattern *P*(***σ***|***h***, ***g***) we obtained, we refered *E*(***σ***|***h***, ***g***) to the energy of the system in Boltzmann distribution.

According to the obtained parameters ***h*** and ***g*** and the function *E*(***σ***|***h***, ***g***), we then assigned an energy value to all potential assemblage patterns. We considered that the assemblage patterns with high energy were unstable and had a low probability of occurring and vice versa.

The Energy Landscape can be constructed once the energy table for all assemblage patterns is obtained. The Energy Landscape was constructed as described in Ezaki’s study [19]. First, the neighbor pattern of assemblage pattern ***σ***, denoted by ***σ***′, was defined as the pattern with only a single assemblage state difference. For example, the assemblage pattern with nine assemblages ***σ*** = (1, −1, −1, −1, −1, −1, −1, −1, −1) and ***σ***′ = (−1, −1, −1, −1, −1, −1, −1, −1, −1) are neighbor patterns to each other since only the first assemblage state is different. We assumed the neighbor patterns are closely related to the original pattern, and the pattern transition to the neighbor patterns was the initial step of any further transitions. Second, the energy of a specific pattern *E*(***σ***) was compared to all its eight neighbor patterns *E*(***σ***′). If the *d*-th neighbor pattern $E(\sigma_{d}^{\prime })$ is the minimum in the comparison, we assumed that the pattern ***σ*** had the closest relation to $\sigma_{d}^{\prime }$, and link them to depict the potential transition direction following the steepest energy descent. Third, once *E*(***σ***) = *E*(***σ***′_*d*_), the pattern *σ* had no other neighbor pattern with lower energy, we defined it as a local minimal pattern (LMP). Intuitively, LMP would be located at the bottom of the energy basin, reflecting the aforementioned transition paths from high energy patterns towards low energy and high stability in the Energy Landscape. Finally, all assemblage patterns belong to one basin through the path linking the pattern to its neighbor pattern and finally reaching the LMP (see the result section). The construction of energy landscape figures was conducted by python NetworkX package [21].

The progression trend of a microbial system can be assumed as starting from an initial assemblage pattern, transiting to its neighbor pattern with higher stability, and repeating the same process towards the LMP with the locally highest stability. The Energy Landscape illustrates the energy relationship of the dynamic microbial system, especially those stable patterns which might contribute to specific health states of the host.

### Code availability

The codes used in this study are stored at github repository: https://github.com/KaiyangZ96/microbiome-energy-landscape.git.

## Results

### LDA modelling result

**Fig 3:**
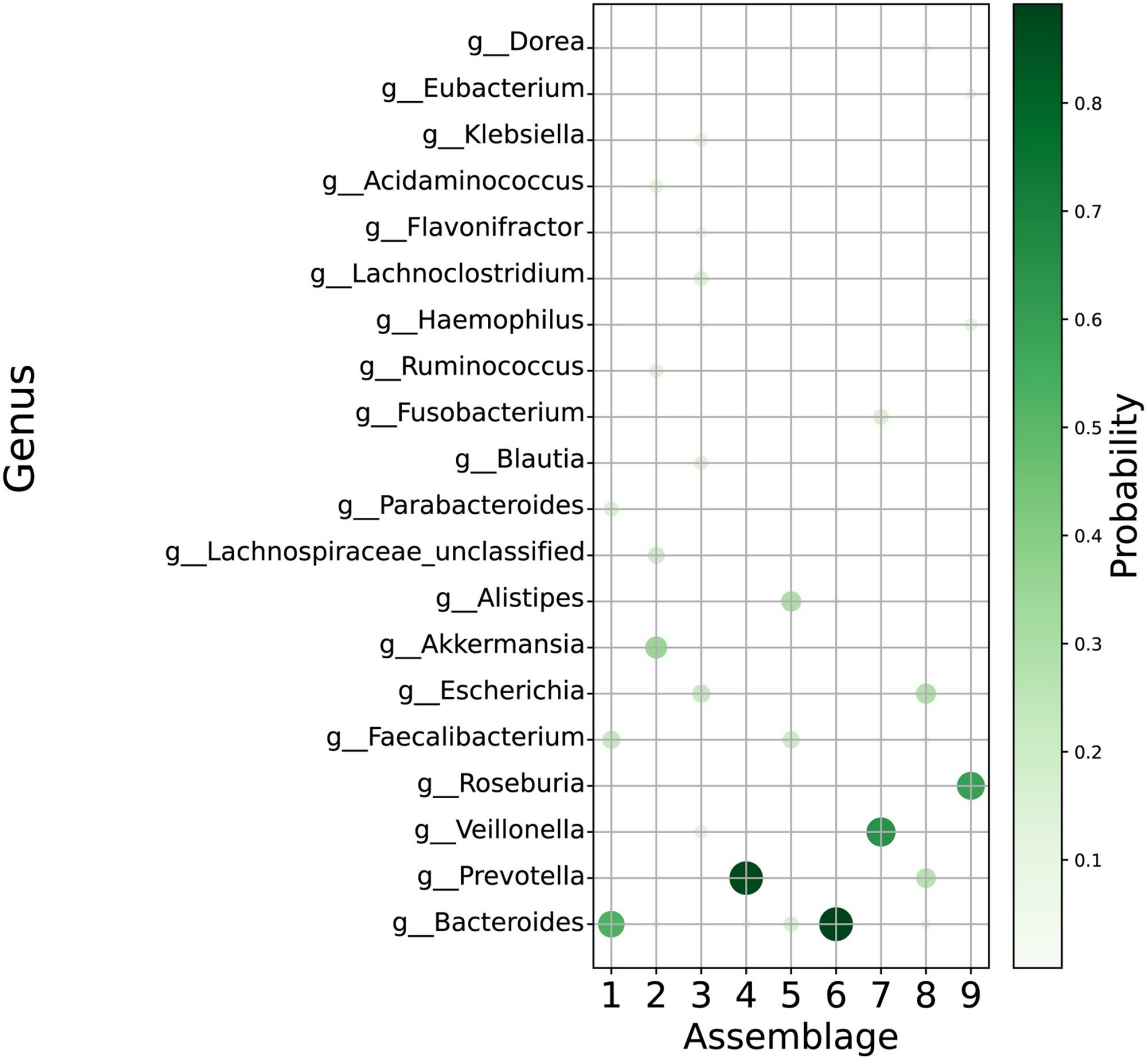
Composition of microbial assemblages derived from LDA model. Figure shows the composition of each assembles ***φ***. Only twenty most frequent genera among all assemblages are depicted.

The parameters ***θ*** and ***φ*** represented the abundance of assemblages and the weight of the components in assemblages, respectively. According to the parameter fitting result, the composition of some assemblages was clearly dominated by a single genus, such as in assemblage #6 and #4 dominated by genus *Bacteroides* (0.89) and *Prevotella* (0.87), respectively. On the other hand, two or more genera mildly dominated others: *Bacteroides* (0.52) and *Faecalibacterium* (0.22) in assemblage #1, *Akkermansia* (0.35) and *Lachnospiraceae* (0.19) in assemblage #2, *Escherichia* (0.22) and *Lachnoclostridium* (0.14) in assemblage #3, *Alistipes* (0.29) and *Faecalibacterium* (0.20) in assemblage #5, *Veillonella*(0.64) and *Fusobacterium* (0.16) in assemblage #7, *Escherichia* (0.29) and *Prevotella* (0.27) in assemblage #8, *Roseburia* (0.60) and *Haemophilus* (0.11) in assemblage #9 (Fig 3, Table 2). The components of one assemblage can be regarded as sharing similar characteristics and contributing jointly to the assemblage’s effects on the host.

**Table 2:** The dominant genera in the assemblages. The values in brackets represent the weight of genera *φ*.

Assemblages	Dominant genera
1	*Bacteroides* (0.52) and *Faecalibacterium* (0.22)
2	*Akkermansia* (0.35) and *Lachnospiraceae* (0.19)
3	*Escherichia* (0.22) and *Lachnoclostridium* (0.14)
4	*Prevotella* (0.87)
5	*Alistipes* (0.29) and *Faecalibacterium* (0.20)
6	*Bacteroides* (0.89)
7	*Veillonella*(0.64) and *Fusobacterium* (0.16)
8	*Escherichia* (0.29) and *Prevotella* (0.27)
9	*Roseburia* (0.60) and *Haemophilus* (0.11)

The abundance of assemblages showed a strong imbalance between the assemblages. In most samples, the abundance of assemblage #6 was notably higher than that of other assemblages (S1B Fig). Although the average abundance of assemblages showed a difference between the three classes (S1A Fig), calculated by ${\left(\theta_{i\;LDAmodeling}\right)}_{average}=\frac{1}{N}\sum_{n=1}^{N}\theta_{in\;LDAmodeling},\text{where}\;N=\left\{\text{CD}:65,\text{UC}:38,\text{non-IBD}:27\right\}$ only included the samples for training the LDA model, there was no assemblage showing a significant difference in abundance between the classes (Kruskal-Wallis Test, *P* > 0.05).

**Fig 4:**
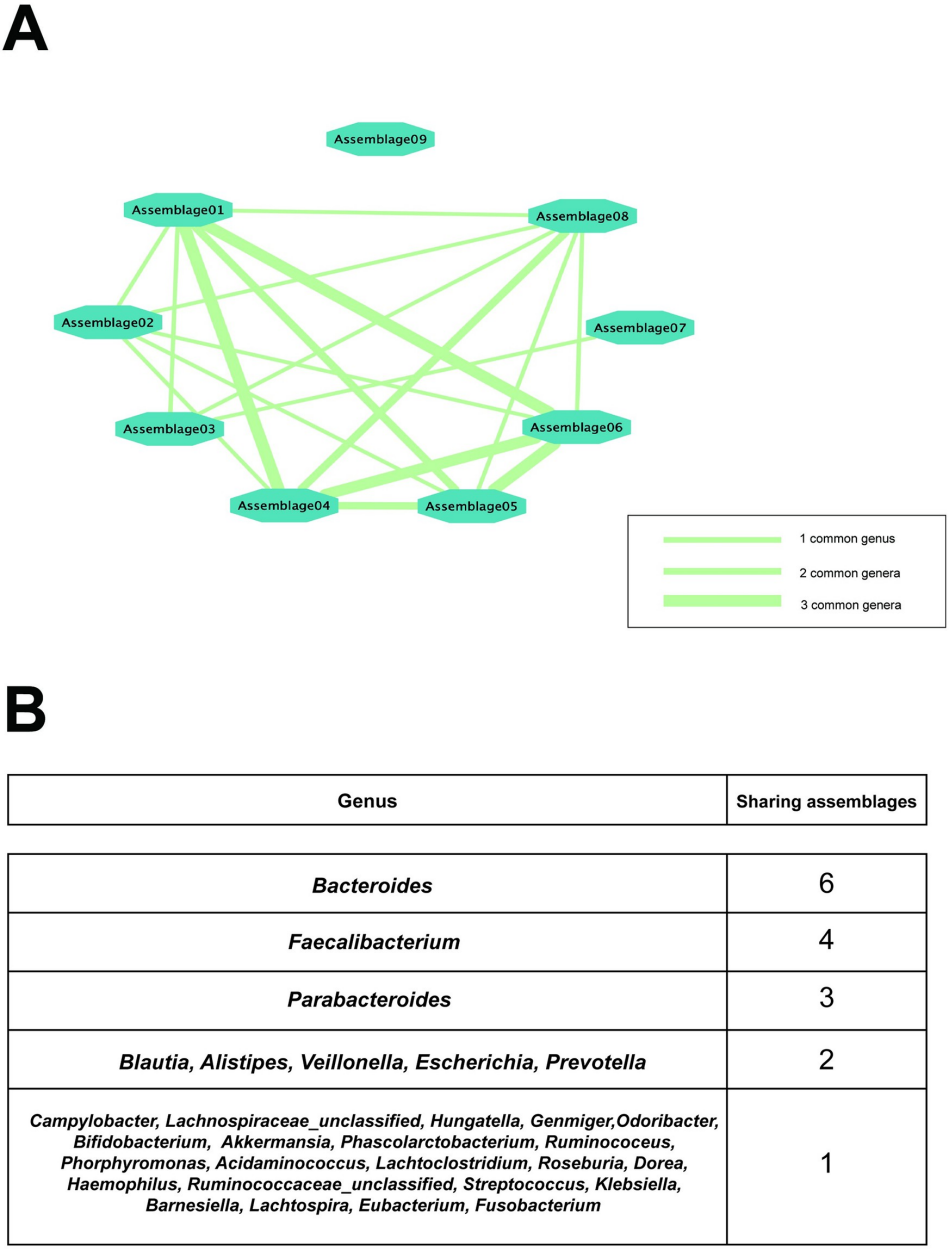
Common genera in the assemblages. **A**: the network shows the relation between assemblages, where the edges are weighted by the common genera number within the top five dominant components of each assemblage. **B**: The table shows the top five dominant genera of all assemblages and the times they recur on different assemblages.

Among the dominant components defined by the top five highest weights in ***φ***, the assemblages shared several common genera. Fig 4A shows the relation between assemblages based on the common dominant genera. Note that assemblages #1, #6, and #4 share three common genera and create a small relation cycle, and only assemblage #9 has no connection to other assemblages. Intuitively, the dominant genera of each assemblage shape the function of the assemblage, so we might learn the functional connection from such a relation network. Fig 4B lists all these dominant genera. The *Bacteroides*, *Faecalibacterium*, and *Parabacteroides* were the most frequent genera, with the six, four, and three folds, respectively, and the other genera were all less than three folds.

After estimating the parameters ***φ***_*i*,*i*∈(1,…,*I*)_, we applied the LDA model to the samples (*N* = 26 × 10 × 3 = 260 × 3 = 780) and obtained the weight of assemblages ***θ*** for following pairwise MaxEnt modeling.

### Pairwise MaxEnt modeling result

**Fig 5:**
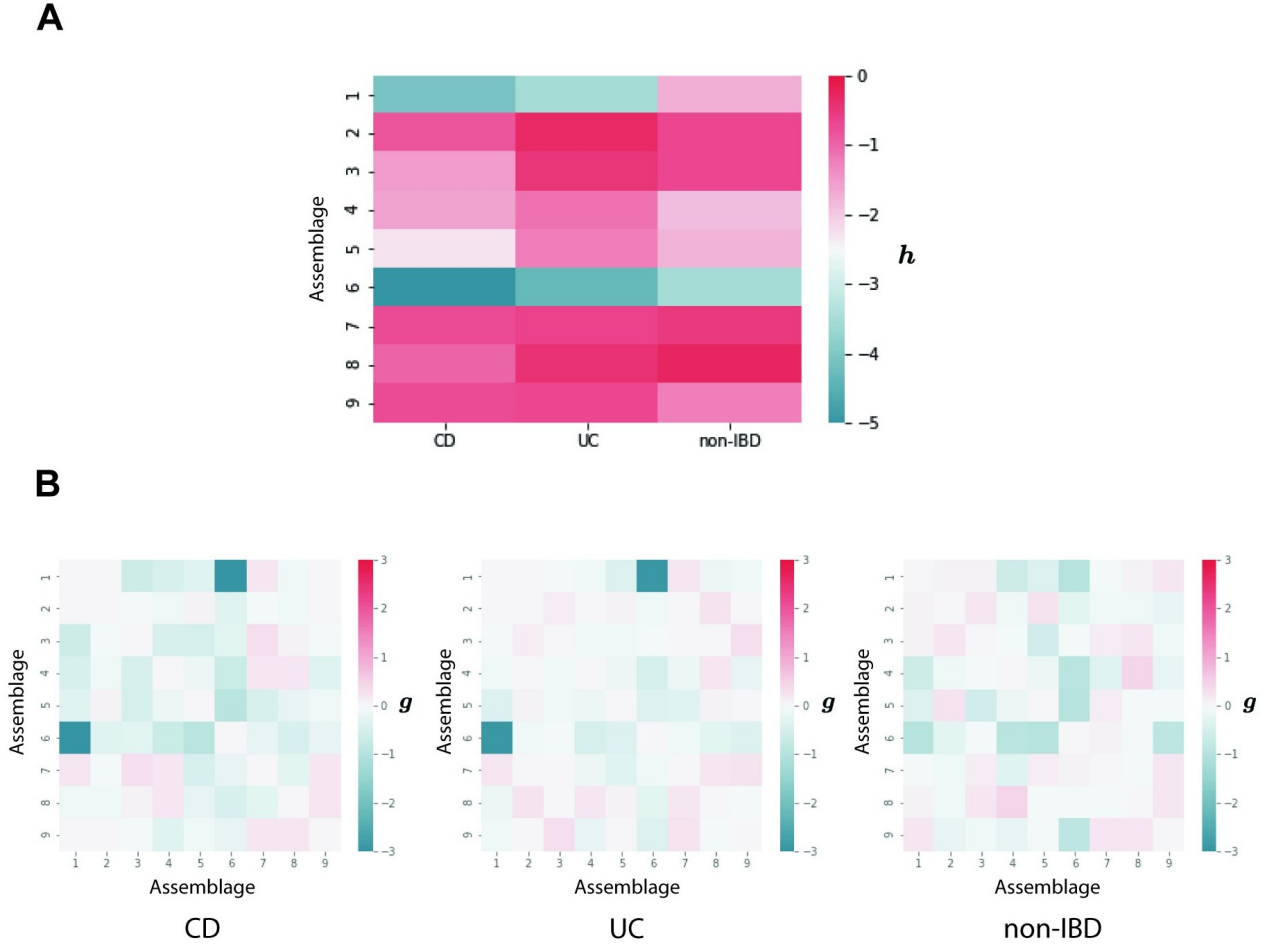
Pairwise MaxEnt results for the three classes. **A**: the heatmap shows the tendency for occurrence of single assemblages ***h*** obtained from the pairwise MaxEnt model. **B**: three heatmaps describe pairwise interactions between assemblages ***g*** obtained from the pairwise MaxEnt model.

The parameters ***h*** and ***g*** were obtained from the pairwise MaxEnt model in the three classes with the same sample size (*N* = 26 × 10 = 260). Fig 5A shows the tendency for the occurrence of single assemblages ***h***. By definition, the low value of ***h*** implies a low energy and high probability of occurring. Notably, the assemblages #1 and #6 dominated by *Bacteroides* had obviously low values than other assemblages. They had lower values in the CD class than in UC and non-IBD classes. Fig 5B shows the pairwise interaction between assemblages. Here the high value of ***g*** in two specific assemblages means their co-occurrence contributes to the low energy of the assemblage pattern. Several differences in interaction features among the three classes can be observed. Interestingly, the value of interaction between assemblages #1 and #6 clearly had a low value in the non-IBD class compared with CD and UC classes.

Besides, the modeling results of other CD classes with different participants showed similar features on both parameters (S2A and S2B Fig), which would support the reproducibility of our method.

### Energy Landscape

**Fig 6:**
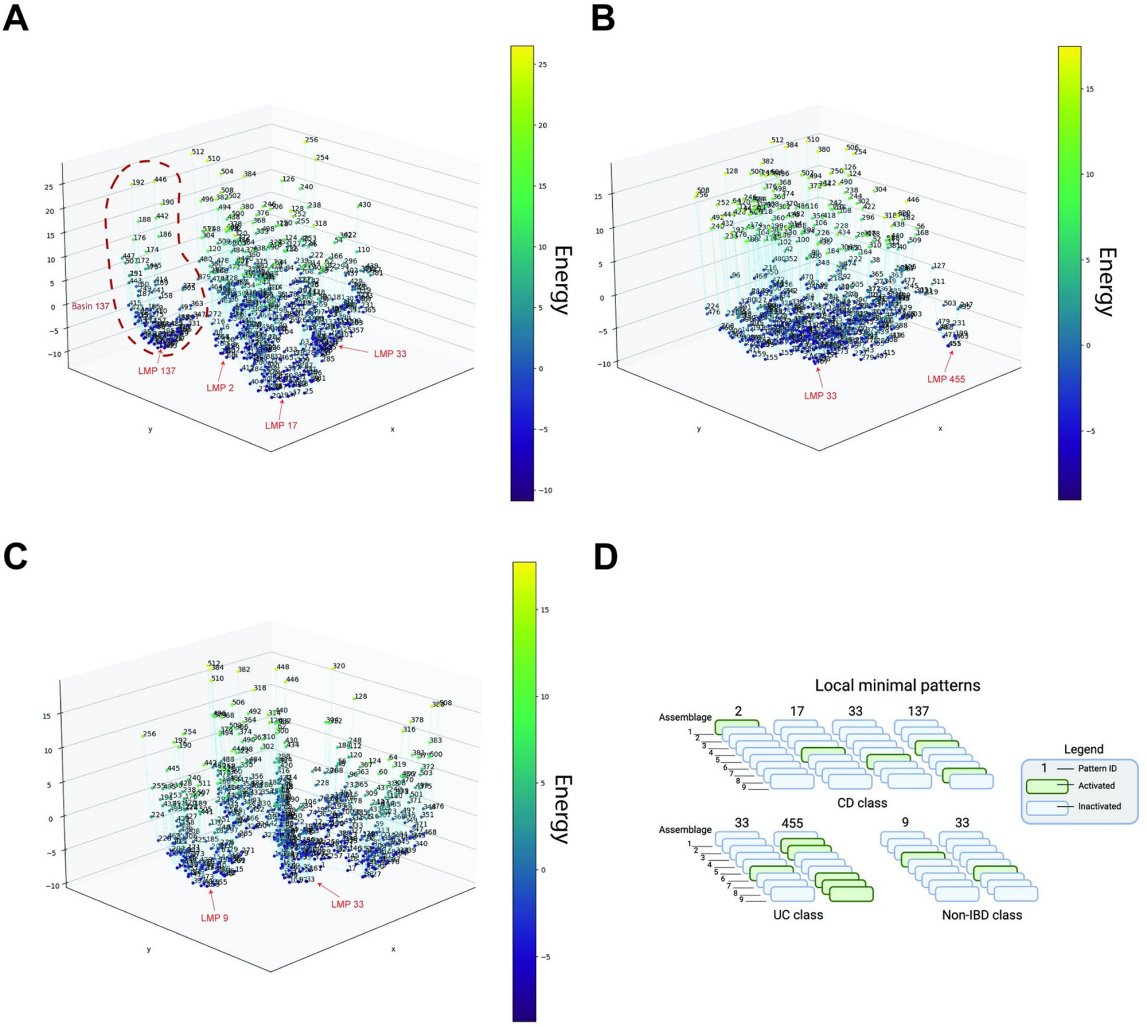
Energy landscape constructed according to the pairwise MaxEnt modeling results. **A, B, C**: the 3D line plot showing the energy of all the patterns of 9 assemblages in CD, UC, and non-IBD class, respectively. Each assemblage pattern is connected to its neighbor pattern with the steepest energy descent or to itself when it is a local minimal pattern. **D**, the composition of each LMP in the three classes: CD, UC, and non-IBD, respectively. The green block means an activated state (+ 1) in the assemblage pattern.

The Energy Landscape was constructed through the energy *E*(***σ***) of 512 assemblage patterns given by the energy function with parameters ***h*** and ***g*** in the methods section. Fig 6A–6C depict the energy of assemblage patterns in the three classes by drawing the line plot linking the patterns with their steepest energy descent neighbor pattern (see the method section 3.6 with energy value as Z-axis, S5 Fig depict the same Energy Landscape in 2D view). Fig 6D shows the LMPs of each class, representing assemblage patterns with locally low energy and high stability. Four LMPs were observed in the CD class: pattern P-#2, pattern P-#17, pattern P-#33, and pattern P-#137, while two LMPs were observed in both UC and non-IBD classes: pattern P-#33 and pattern P-#455 in UC and pattern P-#9 and pattern #33 in non-IBD. Within these LMPs, patterns P-#2, P-#17, P-#33, and P-#9 had only a single positive assemblage, while patterns P-#137 and P-#455 had multiple positive assemblages. Notably, pattern P-#33 was shared in all three classes, and other patterns were unique in specific classes.

From Fig 6A–6C, it can be observed that all the patterns were grouped into small clusters according to the LMP to which they were directed. These clusters can also be regarded as the “energy basins” in the Energy Landscape, which indicate the pattern-shifting trend. Because of the corresponding relation between LMP and energy basin, there were four energy basins in the CD class and two in the UC and non-IBD class. However, unbalanced component size was observed in these energy basins; certain basins were composed of a substantial quantity of patterns, whereas others contained only a few patterns: in the UC class, only 13 patterns were clustered to the basin with LMP P-#455, and the other 499 patterns belonged to the other basin with LMP P-#33.

The energy distribution in three classes is shown in S3 Fig, reflecting the non-identical overall stability of the microbial community. S4 Fig depicts the energy of each sample and the energy variation of each participant in the ten time-series samples, which provides an overall illustration of the energy situation of the participants.

## Discussion

### The potential key bacteria in CD development suggested by the analysis of LMPs

Here, we obtained insights into microbiota alteration when health conditions switch from one to another through the comparison of the LMPs in the energy landscape. As introduced in the method, each LMP represents the local stable assemblage patterns in the view of energy landscape. Collectively, these individual LMPs reflect the global stable stages of the microbiota under specific conditions.

The LMPs were P-#2, P-#17, P-#33, P-#137 in CD, and P-#33, P-#455 in healthy non-IBD class (Fig 6). Interestingly, three assemblages #1, #5 and #6 associated with the genus *Bacteroides* were observed as the only “activated” assemblage in three LMPs— P-#2, P-#17, and P-#33 of the CD class, while only the pattern P-#33 with activated assemblage #6 was in the non-IBD class. According to the probability of *Bacteroides*’ occurrence in assemblages ***φ***_*Bacteroides*_, *Bacteroides* genus was strongly dominant in the assemblage #6 in P-#33 with $\varphi_{Bacteroides}^{assemblage6}=0.89$, mildly dominant in the assemblage #1 in P-#2 with $\varphi_{Bacteroides}^{assemblage1}=0.57$, weakly dominant in the assemblage #5 in P-#17 with $\varphi_{Bacteroides}^{assemblage5}=0.29$. Three *Bacteroides* dominated levels suggest the varied involvement of *Bacteroides* in these stable stages. Alterations in the gut microbiota are strongly associated with the development of IBD, which is characterized by reduced abundance of commensal anaerobic bacteria including members of the *Bacteroides* genus [22–26]. The alteration of *Bacteroides* is also reported in between disease’s phases [23]. Intestinal *Bacteroides* species have evolved a commensal colonization system, contributing to the homeostasis of the gut microbiota [27], and might be attributed to the synthesized conjugated linoleic acid, known for its immunomodulatory properties [6]. However, the longitudinal data with a large sample size and long timescale is yet to show the role of *Bacteroides* in IBD development. Our results support the alternation of *Bacteroides* in the disease development of CD. Besides, the multiple LMPs characterized by different degrees of domination of *Bacteroides* may also highlight *Bacteroides*’s role in shaping the microbiota structure stable patterns in CD, and the alteration of *Bacteroides* might be the key to the transition between these pattrens. If we consider the potential concurrence between the stage of disease development and microbiota, this result also implicates the *Bacteroides* as a potential marker of the disease pathogenesis.

Also among these three LMPs P-#2, P-#17, and P-#33, genus *Alistipes* was the first dominant component in assemblage #5 ($\varphi_{Alistipes}^{assemblage1}=0.29$) of LMP P-#17 apart from the *Bacteroides* in the other two patterns. *Alistipes* has been reported to relate to gut inflammation, but contrasting results about its contribution to the disease have also been reported [28]. Our result may support *Alistipes*’ harmful contribution to CD development, and this contribution might be affected by the decreasing *Bacteroides*.

Interestingly, two genera show different behavior with their previously reported anti-inflammatory property. The genus *Faecalibacterium* was the second dominant component in assemblage #1 of P-#2 ($\varphi_{Faecalibacterium}^{assemblage1}=0.22$) and assemblage #5 of P-#17 ($\varphi_{Faecalibacterium}^{assemblage5}=0.20$). Note that the only species of this genus, *Faecalibacterium prausnitzii*, has been reported to decrease in the IBD pathogenesis [29] and have anti-inflammatory protein production [30]. Besides, Genus *Parabacteroides* was the third dominant component in assemblage #1 of P-#2 ($\varphi_{Faecalibacterium}^{assemblage1}=0.14$). *Parabacteroides spp.* has been identified as a probiotic and related to the alleviation of tumorigenesis and inflammations [31, 32]. Therefore, comparing assemblages #6 of shared LMP P-#33 and #1 of CD specific LMP P-#2, the transition from health pattern P-#33 to disease stable pattern P-#2 with assemblage #1 can be interpreted as the joint effect of three factors: the increase of *Faecalibacterium* and *Parabacteroides*, which are reported beneficial to the disease; the decrease of *Bacteroides*. We could speculate that such “trade-off trend” between these factors from both directions and their contribution to CD development lead to the potential intermediate LMP P-#2 with activated assemblage #1.

Apart from three LMPs associated with *Bacteroides* of CD-specific LMP, in the LMP P-#137 with activated assemblage #4 and assemblage #8, we found *Prevotella* and *Escherichia* as the dominant genus, respectively. Both have been reported to be related to chronic inflammatory disease [33, 34]. We suppose that the concurrence of *Prevotella* and *Escherichia* can be a potential maker of a particular stage in CD development.

We conclude that the aforementioned genera and the interaction of these genera might be the key to the alteration of microbiota in CD development. Especially, the alteration of *Bacteroides* and its “trade-off trend” with other genera are suggested crucial contribution to shaping microbiota stages and facilitating the transition of the stage in the disease development, which remain to be further investigated.

### The methodological advantages of Energy Landscape approach

We combined the LDA model and pairwise MaxEnt model with complementary advantages and achieved the goal of uncovering the hidden microbiota pattern from time-series microbiome data. The LDA model can extract the co-occurrence assemblages from the microbiota [8, 9], however, it doesn’t indicate the stable composition and their transition in the dynamic system. On the other hand, the pairwise MaxEnt model studies the compositional stability of the changing microbiome system [13] but only few high-abundance taxa were selected as input. Our approach combines the two models and incorporates their advantages to assess the global compositional stability of overall microbiota (Table 3).

**Table 3:** The advantages and disadvantages of the two models. The LDA model and pairwise MaxEnt model are complementary to cooperate to study the stability of the dynamic microbiome system. *φ*.

	Advantage	Disadvantage
LDA model	Extract assemblages from microbiota	Not indicate the stability of system
Pairwise MaxEnt model	Study compositional stability	Limited input taxon number

Amos et al. elucidated the gut microbiota structure alteration is specified to the disease stratification and location of IBD showing the heterogeneity of gut microbiota in the IBD development [5]. They used the well-labeled cohort with the collected metadata to compare the alteration of microbiota. Our proposed method showed the consistent result of multiple stable structure patterns under the diseases which might suggest the gut microbiota structure in the intermediate stage of disease development. And those stable structure patterns characterized by *Bacteroides*-associated assemblage show the potential key role of *Bacteroides* in shaping the stages and their transition. Notably, our method gave the result without using the detailed information of patients, which implies the potential to uncover the hidden microbial signatures and their relationship during disease development using the time-series microbiome dataset. This function might enable the exploration of hidden stages in the time-series microbiome data without sufficient descriptive information.

### The technical features surpassing conventional approach

Firstly, our method analyzes the microbiome composition data in a community level and comprehensively considers the complex interactions between the microbial communities. In our proposed approach, the microbial taxonomic group, assemblage, is defined by LDA model based on the abundance co-occurrence and the composition of assemblage considering all the pairwise interactions with both positive and negative directions are analyzed and evaluated by the Maximum Entropy model. In previous studies of the association between the microbiome and IBD, the interactions between species or taxonomic groups are still relatively isolated. Some studies [22, 35, 36] discussed the potential contribution of microbiota in IBD’s development by identifying the significant abundance alteration taxonomic units on the composition data between health and disease cohorts. The joint role between those altered taxa was not to be thoroughly analyzed in these studies. On the other hand, a resent study [37] studied the co-occurrence networks that defined microbial modules ‘quantitative traits’ on IBD development and associated these quantitative traits to genome-wide quantitative trait locus by linkage analysis. Although the co-occurrence network categorized the taxa into community-level modules, the modules were investigated separately without taking their interaction into account.

Secondly, the LDA and pairwise MaxEnt model don’t require independent input, which makes the approach appropriate for the time-series data. In our study, we used the fecal microbiome composition data with 10 successive time points for each participant and separated by a gap of more than one week. Our result is derived from longitude data which should reflect the dynamic characteristics of microbiome alteration. The model enables the researcher to address the association between microbiota and disease from a dynamic perspective. Although IBD, as a chrome disease, is dynamic, microbiome studies have primarily focused on single time points or a few individuals, which makes it hard to capture the dynamic feature of the alteration of the microbiome during the disease pathogenesis. However, the time-series data points on longitude study are dependent and hard to apply to conventional statistical methods for cross-individual comparison requiring the independence of samples. For example, Walker et al. [38] observed the alteration of *Firmicutes* and *Bacteroidetes* in the IBD patients, with the Mann-Whitney U test analysis on single time point microbiome composition data of only a few patients. In Lewis et al.’s study [39], the author explored inflammation and anti-inflammation treatment effects on the composition of the gut microbiota in Crohn’s disease. They analyzed the samples with the comparison of single time points of the microbial composition of health/disease and no-treatment/treatment by quantile regression model.

Thirdly, our method quantitatively describes the probability of the occurrence of all potential combination patterns among bacterial assemblages’ interactions and constructs a global stability view “Energy Landscape” for the homeostasis and dysbiosis of the gut environment. In other words, even the patterns which not occur on the input data will be assigned the energy value given by the parameter estimated from observed data. This feature enables the researcher to analyze and discuss all the situations and observe the transition routes between patterns that represent the intermediate microbiota structure. The prediction of the transition between a current pattern from a sample and its future development might also be available after further improvement of the method. Currently, even though some studies directed attention toward the dynamic of the microbiome, there is a lack of quantitative methods to describe and analyze the absent or rare abundance patterns in the microbiome data of IBD. In Halfvarson et al.’s study [40], although they found the health patients’ microbiome varies within the defined “Health Plane” while the IBD samples are away from the “Health Plane”, only the structures with collected data have been analyzed. Whole potential structural patterns within the IBD development especially those short-term intermediate stages between disease and health, were not to be quantitatively analyzed, which leaves the barrier for studying the shift of microbiome structure from health to disease stage.

In summary, our approach addresses the challenges of conventional microbiota-disease association analysis, through the comprehensive evaluation of interaction between microbial communities, the compatibility to dependent time series data, and the capability to quantitatively analyze all potential patterns of composition. The stable microbiota patterns insight gained from this approach capturing the complex structural and dynamic aspects of gut microbiota in disease development contribute to the growing body of knowledge on microbiota-IBD association.

### Limitations and future work

Considering the function and the role of an assemblage as a unity is challenging. All nine assemblages had their dominant components (Fig 3B), which may be considered to determine the assemblage’s contribution to the host’s microbiome to a great extent. Besides, genera with a much higher probability of occurring in one specific assemblage, or are “unique” in a specific assemblage, will bring special features to the assemblage. However, as observed from the composition of the assemblages, most of the genera satisfy the condition of “unique,” increasing the complexity of studying the function of assemblages. Thus, in this study, we mainly discuss the function of assemblages according to their dominant components. However, a more comprehensive and persuasive method to analyze the assemblages is required for future studies.

Although the potential microbiota structure stages are indicated by the LMPs uncovered from each class, it is still challenging to know the association between them. We speculate the LMP P-#2 in CD is the intermediate stage between healthy pattern P-#33 and more severe pattern P-#17 according to the stepwise change of *Bacteroides* -dominated level in represented assemblages. However, more experimental evidence is necessary to prove their association, and the transition route between the stable patterns merits further discussion in future work.

## Conclusions

In this study, we introduced a novel Energy Landscape approach combining LDA and pairwise MaxEnt models with their complementary benefits to study the heterogeneity of microbiota during the disease pathogenicity from time-series microbiome data. The method uncovers the hidden intermediate microbiota structure and their transition during the microbiome-associated disease’s development and explores the microbial taxa that play key roles in shaping the relevant structures. The analysis with time-series IBD dataset reveals the potential contribution of *Bacteroides* and several genera in CD development. The results demonstrate the method’s promising capability in studying the role of dysbiosis in microbiota-associated diseases.

## Supporting information

S1 Fig The results of LDA modeling and computation. (DOCX)

S2 Fig Application of the pairwise MaxEnt model for two different CD classes. (DOCX)

S3 Fig Energy distribution. (DOCX)

S4 Fig The energy variation in the participants. (DOCX)

S5 Fig Energy landscape in 2D view. (DOCX)
