# Supplementary material for: An energy landscape approach reveals the potential key bacteria contributing to the development of inflammatory bowel disease
Source: PLoS One. 2024 Jun 17;19(6):e0302151. doi: 10.1371/journal.pone.0302151 (PMC11182530; doi:10.1371/journal.pone.0302151)

**S1 Fig. The results of LDA modeling and computation.** **A**: The assemblages’ abundance among three disease classes, compared by the average abundance of the assemblage in each class. The values for each assemblage are scaled to one. **B**: The abundance of assembles among the 130 samples given by the LDA model.


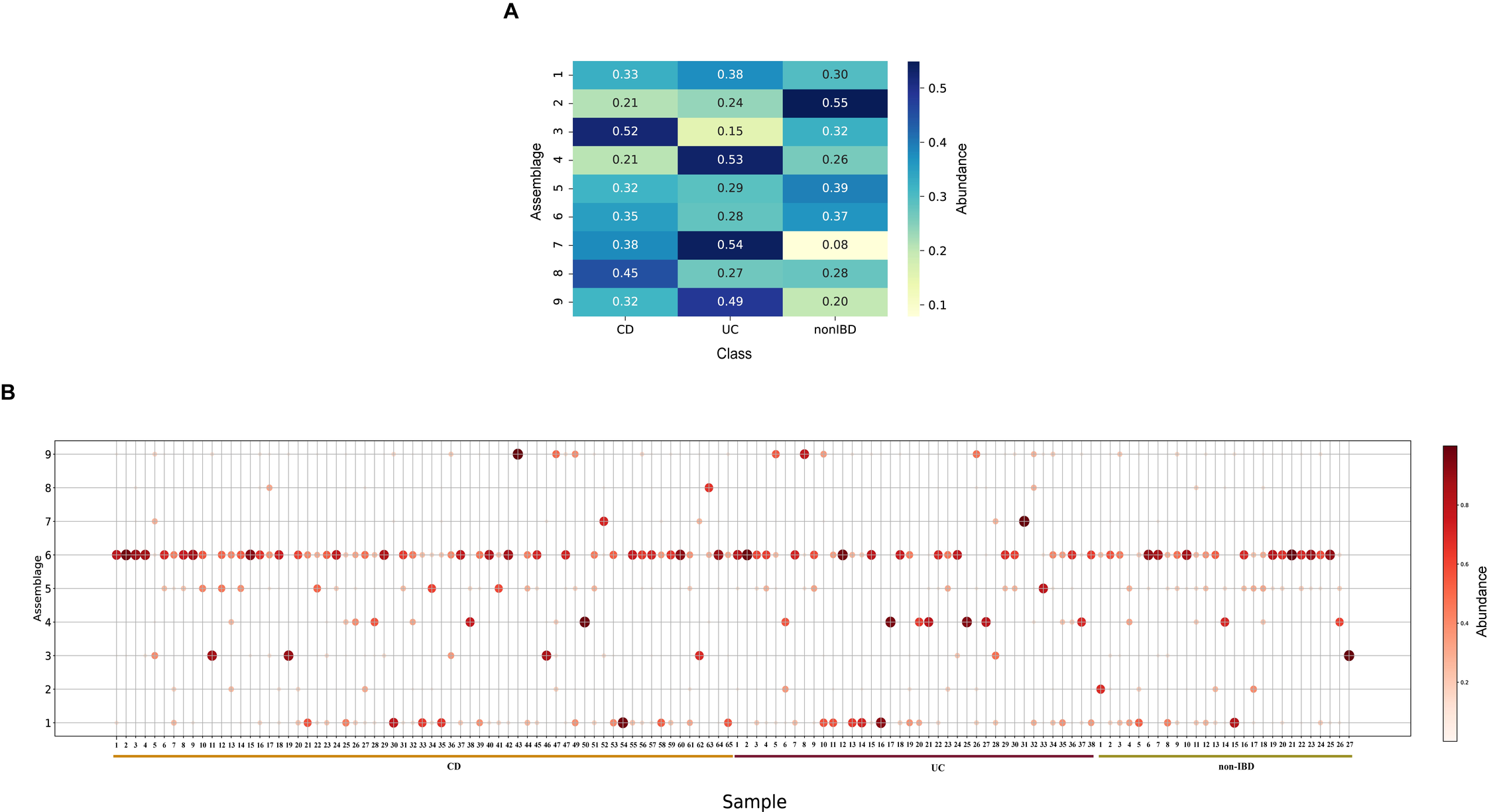

Supplement: S1 Fig — (DOCX) [file pone.0302151.s001.docx]
