# Supplementary material for: An energy landscape approach reveals the potential key bacteria contributing to the development of inflammatory bowel disease
Source: PLoS One. 2024 Jun 17;19(6):e0302151. doi: 10.1371/journal.pone.0302151 (PMC11182530; doi:10.1371/journal.pone.0302151)

**S2 Fig. Application of the pairwise MaxEnt model for two different CD classes. A**: the heatmap showing the tendency to occur of single assemblages 𝒉 obtained from pairwise MaxEnt model. **B**: the heatmap showing pairwise interactions between assemblages 𝒈 obtained from pairwise MaxEnt model.


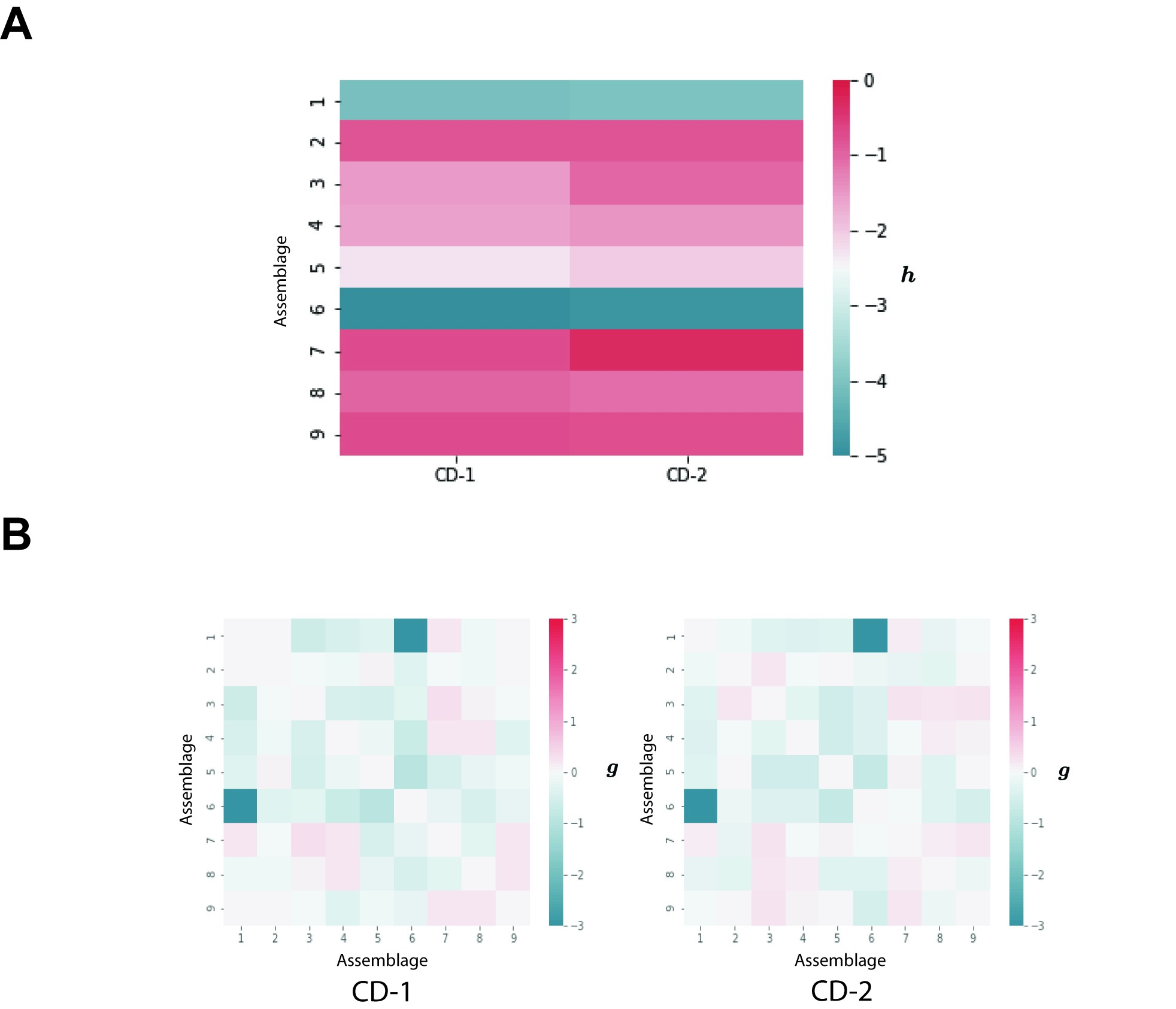

Supplement: S2 Fig — (DOCX) [file pone.0302151.s002.docx]
