# Supplementary material for: An energy landscape approach reveals the potential key bacteria contributing to the development of inflammatory bowel disease
Source: PLoS One. 2024 Jun 17;19(6):e0302151. doi: 10.1371/journal.pone.0302151 (PMC11182530; doi:10.1371/journal.pone.0302151)

**S3 Fig. Energy distribution. A**: the energy distribution among the 512 assemblage patterns in each class. **B**: the energy distribution among the 260 samples in each class.


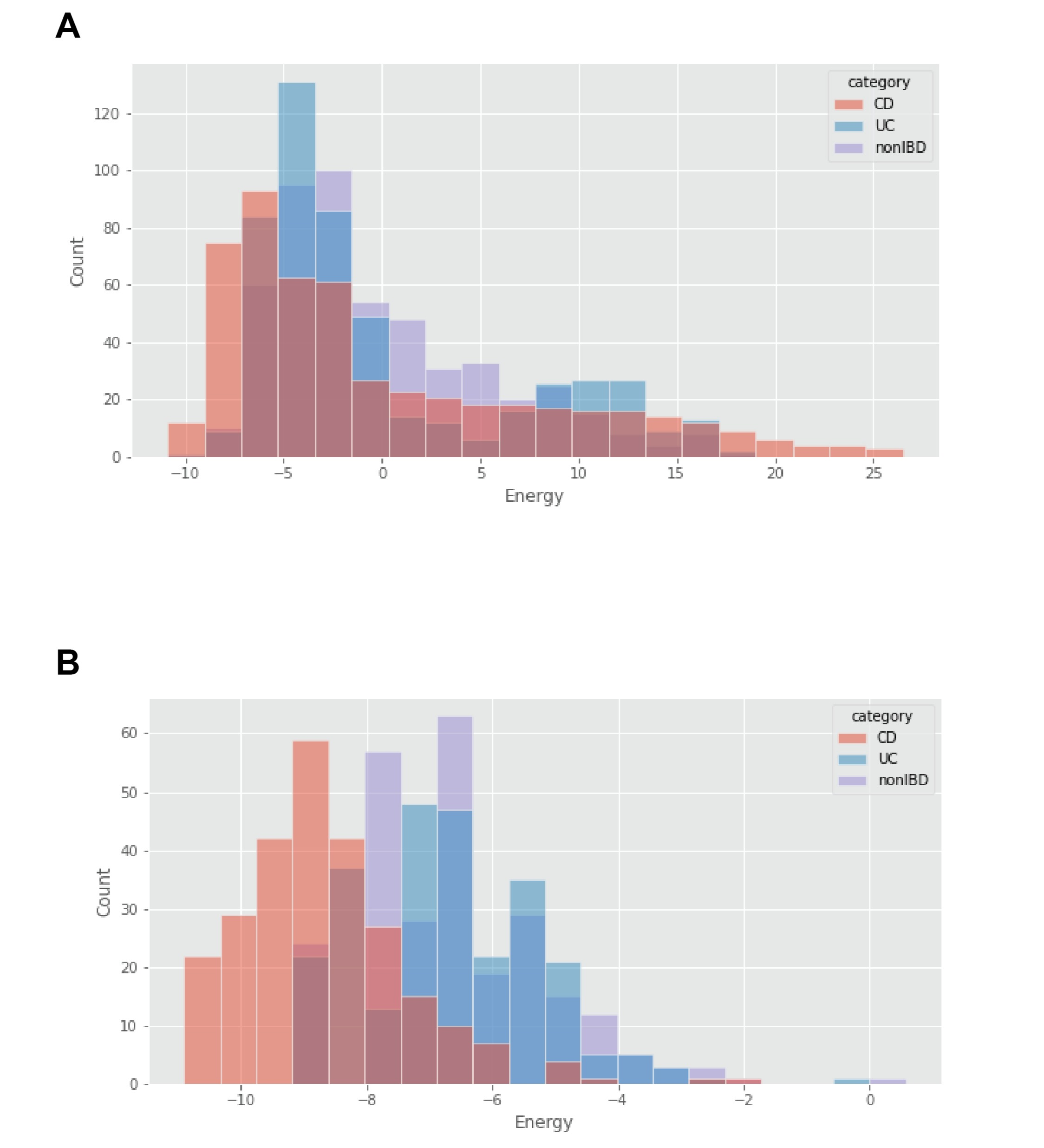

Supplement: S3 Fig — (DOCX) [file pone.0302151.s003.docx]
