# Supplementary material for: An energy landscape approach reveals the potential key bacteria contributing to the development of inflammatory bowel disease
Source: PLoS One. 2024 Jun 17;19(6):e0302151. doi: 10.1371/journal.pone.0302151 (PMC11182530; doi:10.1371/journal.pone.0302151)

**S4 Fig. The energy variation in the participants.** **A, B, C** show the energy value assigned to all samples according to the modeling result in CD, UC, and non-IBD classes, respectively. The lines represent single participants with ten time-series samples.


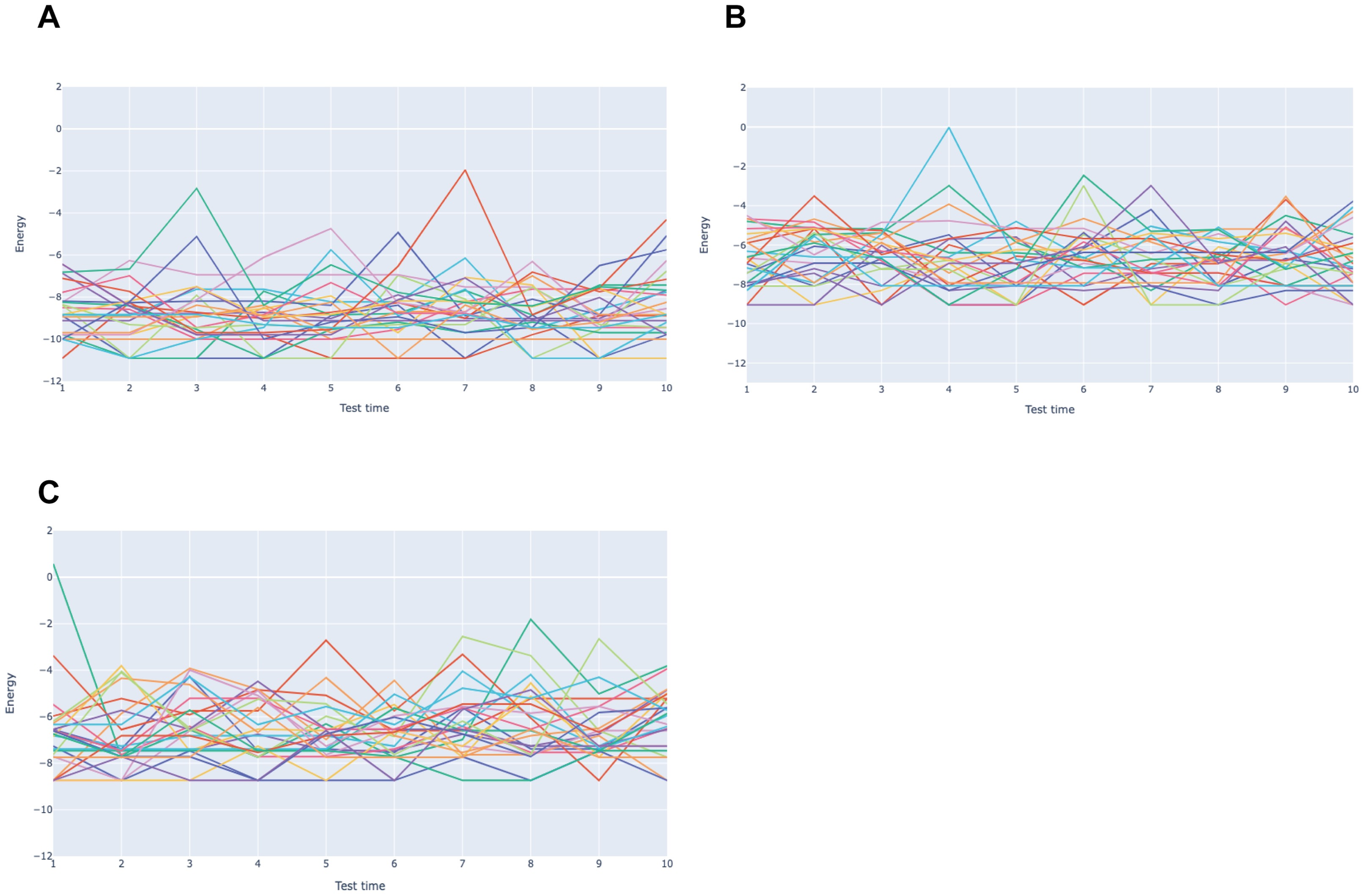

Supplement: S4 Fig — (DOCX) [file pone.0302151.s004.docx]
