# Supplementary material for: An energy landscape approach reveals the potential key bacteria contributing to the development of inflammatory bowel disease
Source: PLoS One. 2024 Jun 17;19(6):e0302151. doi: 10.1371/journal.pone.0302151 (PMC11182530; doi:10.1371/journal.pone.0302151)

**S5 Fig. Energy landscape in 2D view. A, B, C**: the 2D line plot showing the energy of all the patterns of 9 assemblages in CD, UC, and non-IBD classes. The red circles indicate the LMPs.


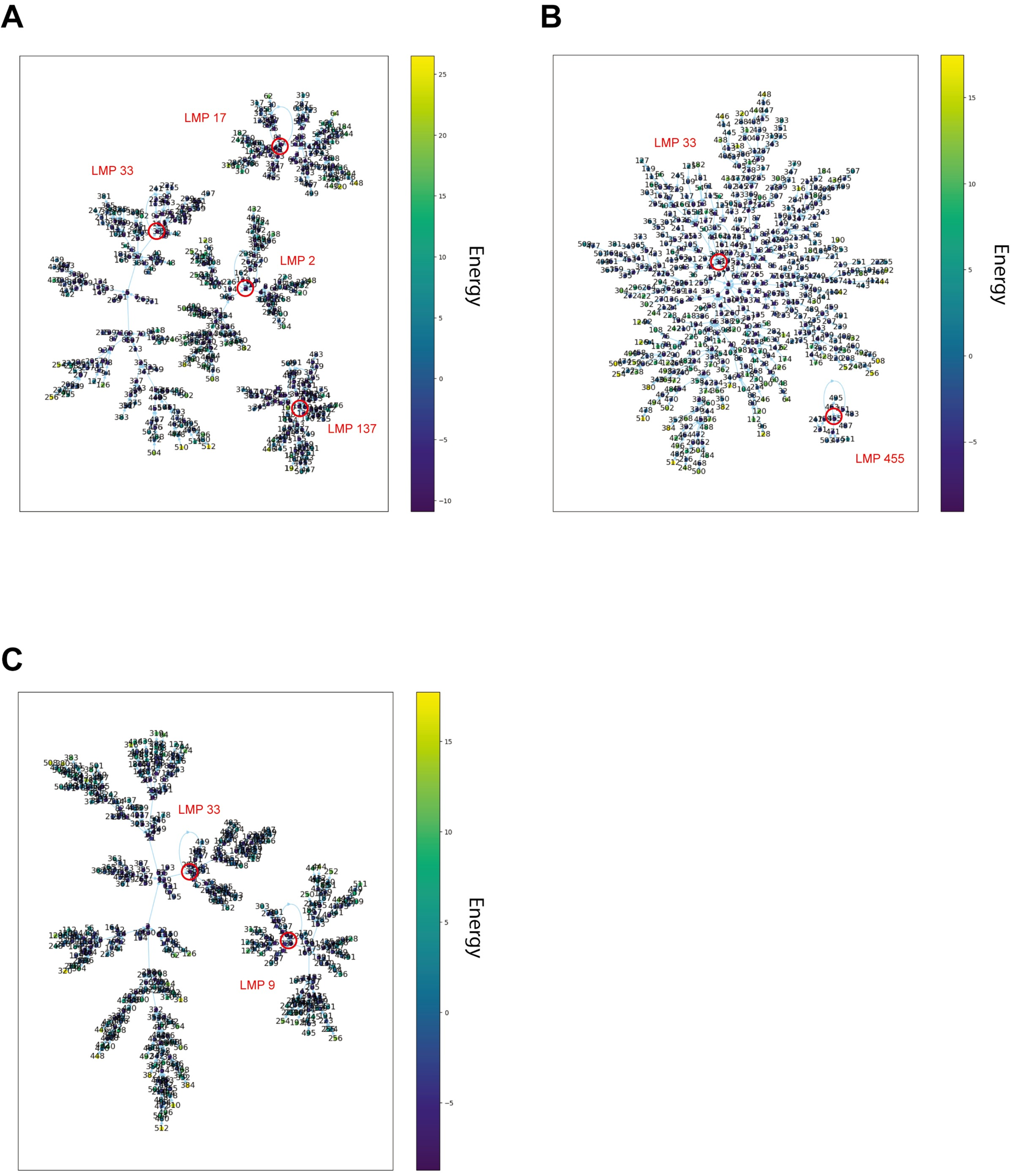

Supplement: S5 Fig — (DOCX) [file pone.0302151.s005.docx]
